# The Type III Effector NopM from *Bradyrhizobium elkanii* USDA61 Induces a Hypersensitive Response in *Lotus japonicus* Root Nodules

**DOI:** 10.1264/jsme2.ME25020

**Published:** 2025-10-15

**Authors:** Cui Ying, Satomi Nozawa, Shohei Kusakabe, Pongpan Songwattana, Pongdet Piromyou, Pakpoom Boonchuen, Panlada Tittabutr, Nantakorn Boonkerd, Hisayuki Mitsui, Shusei Sato, Neung Teaumroong, Shun Hashimoto

**Affiliations:** 1 Graduate School of Life Science, Tohoku University, 2–1–1 Katahira, Aoba-Ku, Sendai 980–8577, Japan; 2 School of Biotechnology, Institute of Agricultural Technology, Suranaree University of Technology, 111 University Avenue, Suranaree, Muang, Nakhon Ratchasima 30000, Thailand

**Keywords:** NopM, *Lotus japonicus*, symbiosis, infected nodule cell, immune signaling

## Abstract

Leguminous plants establish root nodule symbiosis, which is initiated by the recognition of rhizobial nodulation factors by plant receptor kinases. However, other factors, such as Type III effector proteins, also affect host specificity. We herein investigated the role of nodulation outer protein M (NopM), a Type III effector of *Bradyrhizobium elkanii* USDA61, in symbiosis with *Lotus japonicus* MG-20 and *Lotus burttii*. NopM, annotated as an E3 ubiquitin ligase, triggers an early senescence-like response, inducing brown nodules that hinder effective symbiosis. NopM shares structural features with E3 ubiquitin ligases derived from both pathogenic and symbiotic bacteria, including a leucine-rich-repeat and E3 ubiquitin ligase domain. The deletion of these domains or substitution of the cysteine residue, predicted to be the active site of the ubiquitin ligase domain, suppressed the formation of brown nodules. These results suggest that NopM interacts with target proteins through its leucine-rich-repeat domain and mediates ubiquitination via its ligase domain, thereby contributing to the induction of brown nodules. A transcriptome ana­lysis further suggested that the early senescence-like response closely resembled the plant hypersensitive response, with the up-regulation of defense-related genes. Therefore, *L. japonicus* may recognize NopM in infected nodule cells, leading to an immune response that disrupts symbiosis. The present study provides insights into the mole­cular mechanisms by which rhizobial effectors modulate symbiotic interactions in infected nodule cells, highlighting the ability of *L. japonicus* to activate immune responses even in nodule cells where rhizobia have been accepted.

Legumes establish symbiotic relationships with rhizobia, a group of nitrogen-fixing bacteria. This interaction leads to the formation of root nodules in leguminous plants, in which rhizobia reduce nitrogen to ammonia and supply it to the host plants. In return, the host plants provide rhizobia with organic compounds derived from photosynthesis, creating a mutually beneficial partnership (Atkins, 1984; White *et al.*, 2007).

Symbiosis between leguminous plants and rhizobia begins with the recognition of organic compounds, such as flavonoids and phenolic acids, which are exuded from the roots of the host plants by rhizobia through the transcription factor NodD (Shimamura *et al.*, 2022). Upon the recognition of these organic compounds, NodD activates the expression of nodulation genes, leading rhizobia to synthesize and secrete nodulation factors (NFs). The basic structure of NFs is a lipochitooligosaccharide with a long-chain fatty acid attached. There is diversity in the structure, such as the degree of chitin polymerization, the length of the long-chain fatty acid, and chemical modifications, depending on the species of rhizobia (Haeze and Holsters, 2002). Leguminous plants strictly recognize the structures of compatible NFs through the receptor kinases NFR1 and NFR5, initiating the acceptance of rhizobial infection and the organogenesis of root nodules (Radutoiu *et al.*, 2003, 2007). Some rhizobia secrete a cocktail of NFs with diverse structures, which induce nodulation in various legume species through NFR signaling. Such rhizobia, including *Rhizobium* sp. NGR234, *Sinorhizobium fredii* HH103, and *Bradyrhizobium elkanii* USDA61, are referred to as broad-host-range rhizobia (Carlson *et al.*, 1993; Pueppke and Broughton, 1999).

While NFs are the primary factors initiating symbiosis, some rhizobia also employ the Type III secretion system (T3SS) to further modulate host responses. Similar to the Nod gene cluster, the expression of T3SS is induced by the transcriptional regulator NodD. T3SS is a secretion system that is widely conserved in Gram-negative bacteria, utilizing a needle-like structure to deliver effector proteins, known as type III effectors (T3Es), into target cells. Rhizobia use T3SS to inject effector proteins into legume hosts, manipulating host cell signals to promote infection and nodulation (Teulet *et al.*, 2019; Ratu *et al.*, 2021). However, depending on the host-rhizobia combination, these effectors may sometimes trigger host defense responses, suppressing nodulation signals initially activated by NFs and preventing the establishment of symbiosis (Sugawara *et al.*, 2018; Hashimoto *et al.*, 2020; Kusakabe *et al.*, 2020). Many rhizobial T3Es involved in symbiosis have been identified; however, their functions, as well as the mechanisms by which host plants recognize and respond to them, remain largely unknown.

We previously identified Nodulation Outer Protein M (NopM) as a T3E of *B. elkanii* USDA61 that induces symbiotic incompatibility with *Lotus* spp. The *Lotus japonicus* MG-20 accession and *L. burttii* recognize the NFs produced by USDA61, allowing the rhizobial infection of root tissues and the progression of the symbiotic process up to the formation of nodule primordia. However, upon the presence of NopM from USDA61, the symbiotic interaction is disrupted, resulting in the formation of small, incomplete nodules with a brownish appearance (Kusakabe *et al.*, 2020). These NopM-induced brownish nodules exhibit morphological characteristics similar to senescent nodules. Since this response occurs at an early stage of symbiosis, it has been defined as an early senescence-like response (Kusakabe *et al.*, 2020). While previous studies on the T3Es of rhizobia have primarily focused on their roles in the initial infection process, their functions after rhizobia are released into nodule cells remain unclear. The present study addresses this gap by investigating the role of NopM in these specialized cells, where rhizobia are accommodated within the host. This approach provides novel insights into the immune responses of nodule cells and the mole­cular mechanisms underlying T3E-mediated plant-microbe interactions.

NopM of *B. elkanii* USDA61 was annotated as an E3 ubiquitin ligase containing an N-terminal leucine-rich repeat (LRR) domain and C-terminal ligase domain (Kusakabe *et al.*, 2020). Similar structural features are found in pathogenic bacterial effectors, such as IpaH7.8 of *Shigella* and SspH2 of *Salmonella*, which manipulate host cellular processes via targeted ubiquitination (Quezada *et al.*, 2009; Suzuki *et al.*, 2014). These parallels suggest that NopM also functions through host protein ubiquitination to modulate symbiotic interactions. Through its LRR domain, IpaH7.8 interacts with target proteins in macrophages and ubiquitinates them via a cysteine residue in its ligase domain, which serves as the active site, leading to macrophage cell death and facilitating bacterial infection (Suzuki *et al.*, 2014). Similarly, the E3 ubiquitin ligase SspH2 from *Salmonella* has been reported to localize to the apical membrane of polarized epithelial cells (the membrane facing the external environment) via its N-terminal domain containing an LRR. A model has been proposed in which site-specific ubiquitination within host cells contributes to the successful infection of the host (Quezada *et al.*, 2009).

In addition to pathogenic bacteria, some rhizobia have also been reported to possess NopM, and part of its function in root nodule symbiosis has been elucidated. The type III effector NopM from *Sinorhizobium* sp. strain NGR234 contains conserved LRR and ligase domains, and the cysteine residue in the ligase domain was shown to be essential for‍ ‍both ligase activity and increasing nodule formation on‍ ‍*Lablab purpureus* roots (Xin *et al.*, 2012). Moreover, when NopM from NGR234 is expressed in the roots of *L.‍ ‍japonicus* MG-20 via transformation, infection by *Mesorhizobium japonicum* is promoted (Wang *et al.*, 2024); however, the weight of nodules is reduced (Xu *et al.*, 2018). This reduction caused by the introduction of NopM from NGR234 was shown to be abolished by substituting the cysteine residue at position 338 with alanine (Xu *et al.*, 2018). These findings indicate that the ligase activity of NGR234 NopM, mediated by its catalytic cysteine residue, affects root nodule symbiosis in a legume host-dependent manner. Nevertheless, many aspects remain unclear, such as the importance of the LRR domain and the specific physiological effects of NopM on legume hosts.

In the present study, we focused on elucidating the function of NopM in *B. elkanii* USDA61 to induce the early senescence-like response in *L. japonicus* MG-20 and *L. burttii*, and also investigated the physiological phenomena underlying this response. Since bacterial E3 ubiquitin ligases, such as IpaH7.8 of *Shigella* and SspH2 of *Salmonella*, manipulate host immune responses through ubiquitination, we hypothesized that NopM of *B. elkanii* USDA61 similarly targets host proteins to modulate symbiosis. Specifically, we proposed that NopM-mediated ubiquitination triggers an immune response resembling early nodule senescence in *Lotus* spp. To test this hypothesis, we generated domain deletion and amino acid substitution mutants of USDA61 NopM and exami­ned their symbiotic phenotypes in *L. japonicus* MG-20 and *L. burttii*. We then investigated the symbiotic phenotypes with *M. japonicum* on *L. japonicus* roots transformed with constructs in which NopM or its mutants (domain deletion and amino acid substitution variants) were expressed under the control of the leghemoglobin promoter. Furthermore, to elucidate the mole­cular mechanism underlying the early senescence-like response, we performed RNA-seq on the roots and nodules of *L. japonicus* MG-20 inoculated with the USDA61 wild-type strain or *nopM* knockout mutant. The results obtained revealed that the LRR domain and the cysteine residue at position 402 in the ligase domain of NopM were essential for the induction of brownish nodules in *L. japonicus*. Moreover, a transcriptome ana­lysis strongly suggested that the physiological phenomenon previously defined as an early senescence-like response corresponded to a hypersensitive response (HR).

## Materials and Methods

### Bacterial strains and growth conditions

The bacterial strains and plasmids used in the present study are listed in Table 1. *B. elkanii* USDA61 and its derivatives were grown at 28°C in yeast-mannitol (YM) (Giraud *et al.*, 2000) or arabinose-gluconate (AG) medium (Sadowsky *et al.*, 1987) or HEPES-MES salt medium (Cole and Elkan, 1973) supplemented with arabinose (0.1% w/v) and yeast extract (0.25% w/v). *Escherichia coli* strains were grown at 37°C in Luria–Bertani medium. When required for mutant strain construction, sucrose (10% w/v), kanamycin (200‍ ‍μg mL^–1^), or polymyxin B (50‍ ‍μg‍ ‍mL^–1^) was added to the medium.

### Construction of mutant strains

To generate domain deletion mutants of *B. elkanii* USDA61, approximately 1,000-bp sequences upstream and downstream of the target regions were amplified by PCR using specific primers (Table 2) and cloned into SmaI-linearized pK18mobsacB (Schäfer *et al.*, 1994) via In-Fusion cloning. The resulting plasmids were designated pK18mobsacB_Ligase1, pK18mobsacB_Ligase2, and pK18mobsacB_LRR, respectively (Table 1).

Regarding the amino acid substitution mutant, which introduces a cysteine-to-alanine change at amino acid position 402, the *nopM* coding region along with approximately 700 bp of its upstream and downstream flanking sequences were amplified from USDA61 genomic DNA using specific primers (Table 2). These fragments were cloned into SmaI-linearized pK18mobsacB by In-Fusion cloning to construct pK18mobsacB–NopM±700 bp (Table 1). The amino acid substitution was then performed on this plasmid using the site-directed mutagenesis method (Takara). Outward PCR amplification was performed using mutagenic primers (Table 2) with 15-bp overlaps at the 5′ ends, using pK18mobsacB–NopM±700 bp as a template, and the PCR product was transformed into *E. coli* DH5α. Plasmids were extracted from the resulting colonies, and the mutated region was confirmed by Sanger sequencing. The plasmid containing the desired mutation was designated as pK18mobsacB–NopM (C402A)±700 bp (Table 1).

*E. coli* harboring each plasmid for domain deletion and amino acid substitution was mixed with the helper strain *E. coli* pRK2013 (Figurski and Helinski, 1979) and USDA61 to introduce the plasmid into USDA61 via tri-parental mating. Single-crossover recombinants of USDA61 were selected based on kanamycin resistance (200‍ ‍μg mL^–1^) and sucrose sensitivity (10%), while double-crossover recombinants were selected for kanamycin sensitivity and sucrose resistance. In the strains obtained, the mutated regions were confirmed by Sanger sequencing, and strains carrying the desired mutations were designated Ligase 1, Ligase 2, LRR, and C402A (Table 1).

### Introduction of His-tagged NopM and its variants into USDA61

To introduce His-tagged NopM and its variants into USDA61, primers were designed to amplify approximately 500 bp upstream from the *nopM* start codon and the NopM coding sequence with a C-terminal His×6 tag (Table 2). Using this primer set, PCR was performed with genomic DNA from wild-type USDA61 and the respective domain deletion mutants as templates to obtain DNA fragments of upstream500bp-NopM-His, upstream500bp-Ligase 1-His, upstream500bp-Ligase 2-His, and upstream500bp-LRR-His. The PCR products were cloned into pK18mobsacB by an infusion‍ ‍reaction, and the resulting plasmids were designated pK18mobsacB_Ligase1_His, pK18mobsacB_Ligase2_His, and pK18mobsacB_LRR_His (Table 2). According to the tri-parental mating and screening methods described above, single recombinant clones of USDA61 harboring these plasmids were obtained. Correct integration was confirmed by PCR and Sanger sequencing, and the confirmed strains were used in subsequent culture supernatant ana­lyses.

### Analysis of proteins in the culture supernatant

To investigate the secretion of NopM and its derivatives, *B. elkanii* and its mutant strains (Ligase 1, Ligase 2. LRR, NopM-His, Ligase-1-His, Ligase-2-His, and LRR-His) were inoculated into 120‍ ‍mL AG medium at a 1:100 dilution from the precultures and supplemented with 10‍ ‍μM genistein (dissolved in methanol) to induce T3SS expression. Cultures were incubated at 200‍ ‍rpm at 28°C for 48 h, and the culture supernatant was sequentially centrifuged at 4,000×*g* at 4°C for 1‍ ‍h to remove bacterial cells, followed by further centrifugation at 8,000×*g* at 4°C for 30‍ ‍min to remove the remaining debris. The resulting supernatant was used for protein extraction. Proteins were extracted using the phenol-methanol method (Kusakabe *et al.*, 2020), with modifications in the precipitation and washing steps as described below. The culture supernatant was mixed with Tris-EDTA-saturated phenol, followed by centrifugation at 10,000×*g* at room temperature for 30‍ ‍min to separate the phenol and aqueous phases. The phenol phase was collected, combined with methanol containing ammonium acetate, and incubated at –20°C overnight. Precipitated proteins were recovered by centrifugation at 10,000×*g* at 4°C for 1 h, washed twice with 70% ethanol, air-dried at room temperature for 10‍ ‍min, and resuspended in 8 M urea and 2% w/v CHAPS. Protein concentrations were measured using the Bradford assay (Bio-Rad), and samples were stored at –80°C for further ana­lyses.

Extracellular proteins (5‍ ‍μg of total protein per lane) were separated on 10% SDS-PAGE gels (ATTO) using a constant current of 25 mA for 1‍ ‍h (Bio-Rad Power Pac 1000). Gels were stained with Coomassie Brilliant Blue R-250 and destained in 40% methanol and 10% acetic acid to visualize total protein bands. In the Western blot ana­lysis, proteins (5‍ ‍μg per lane) were semi-dry transferred onto PVDF membranes (90‍ ‍min, 60 V), blocked with 2% BSA in 0.1% Tween-20 PBS at room temperature for 1 h, and incubated with an anti-His antibody with HRP (1:20,000; GE Healthcare) at‍ ‍room temperature for 1 h. Signals were detected using the ChemiDoc^TM^ XRS+ imaging system (Bio-Rad) following an incubation with chemiluminescence substrates (PerkinElmer).

### Root transformation

To construct plasmids for introducing NopM into *L. japonicus* roots, PCR amplification was performed to obtain fragments of NopM and its variants using genomic DNA from *B. elkanii* USDA61 and its domain deletion mutants as templates. Amino acid substitutions were introduced using site-directed mutagenesis primers. To generate the C402A substitution mutant, two PCRs were performed using genomic DNA from USDA61 as the template, with the primer pairs LB3pro_nopM61_F/NopM61_CtoA_R and NopM61_CtoA_F/LB3ter_nopM61_R (Table 2). The resulting PCR products were joined by overlap PCR to obtain the NopM fragment carrying the C402A substitution. The backbone vector for transformation was based on pUB-GFP. To replace the ubiquitin promoter and terminator with the *L. japonicus* Leghemoglobin 3 (LB3) promoter and terminator, inverse PCR was performed to generate a linear vector lacking the original regulatory elements. The LB3 promoter and terminator were amplified by PCR using pCambia_LB3promoter_GW_LB3terminator (GFP) (Shimoda *et al.*, 2019) as the template. The primers used in these experiments are listed in Table 2. The inserts and vector were assembled using an In-Fusion reaction to construct plasmids carrying *nopM* or its variants under the control of the LB3 promoter and terminator. These plasmids were designated as pLB3-NopM, pLB3-Ligase1, pLB3-LRR, and pLB3-C402A (Table 1).

The constructed plasmids were introduced into *Rhizobium rhizogenes* AR1193 and used for *L. japonicus* root transformation. Seedlings were excised horizontally at the hypocotyl and placed on an LB agar plate containing *R. rhizogenes* AR1193. After an incubation at 25°C in the dark for 3–5 days in a humid chamber, they were transferred to 1/2 B5 medium containing meropenem. Transformed roots were selected 14–20 days post-inoculation based on GFP fluorescence using a stereo fluorescence microscope (SZX12; Olympus).

### Nodule phenotype ana­lysis

*L. japonicus* MG-20 and *L. burttii* were used in the nodule phenotype ana­lysis. Seeds were surface sterilized by immersion in 1‍ ‍mL sulfuric acid for 10‍ ‍min, followed by five rinses with sterilized water. They were then treated with 0.2% (v/v) sodium hypochlorite and 0.1% (v/v) Tween-20 for 40‍ ‍min, followed by five additional rinses with sterilized water. Seeds were hydrated in sterilized water for 5‍ ‍h and germinated on 0.8% (w/v) agar plates at 28°C in the dark for 2–3 days. Germinated seedlings were transplanted into autoclaved vermiculite pots containing 300‍ ‍mL nitrogen-free BNM medium (Ehrhardt *et al.*, 1992), with nine seedlings per pot. *B. elkanii* USDA61 and its derivatives were cultured in YM medium at 28°C with shaking for 3–5 days until an OD_600_ of approximately 4.0 was reached. Cells were harvested by centrifugation at 8,000×*g* at room temperature for 2‍ ‍min, washed three times with sterilized water, and resuspended in nitrogen-free BNM medium. Each seedling was inoculated with 1‍ ‍mL of the bacterial suspension (OD_600_=0.1). Plants were grown in a growth chamber at 28°C with a 16-h light/8-h dark photoperiod. Nodule numbers and phenotypic characteristics (color and size) were observed 4 weeks post-inoculation.

Plants with transgenic roots selected based on GFP fluorescence were transferred to pots, inoculated with *M. japonicum* MAFF303099, and grown under the same cultivation conditions as those described above. Nodule symbiotic phenotypes were observed 4–5 weeks after the inoculation.

### RNA sequencing (RNA-seq)

RNA-seq was performed on nodule samples, including associated roots, from *L. japonicus* MG-20 plants inoculated with either the *B. elkanii* USDA61 wild type or a NopM-deficient mutant. Regarding each biological replicate (*n*=4), a sample of a root with a nodule 4 weeks post-inoculation (the time point at which brown coloration of the nodules was observed) was collected from 4 to 6 individual plants, pooled, immediately frozen in liquid nitrogen, and stored at –80°C until RNA extraction. Total RNA was extracted using an RNeasy Plant Mini Kit (Qiagen). The concentration and purity of RNA were measured using a NanoDrop spectrophotometer (Thermo Fisher Scientific) and RNA integrity was assessed using an Agilent 2100 Bioanalyzer (Agilent Technologies). In each sample, 1,000‍ ‍ng of total RNA was used for rRNA depletion using an Ribo-Zero rRNA Removal Kit (Illumina), and RNA-seq libraries were constructed with TruSeq RNA Sample Prep Kit v2-Set A (Illumina). Sequencing was performed on a NextSeq 500‍ ‍system (Illumina) using the 75-bp single-end strategy. The sequencing service was provided by the Kazusa DNA Research Institute, Chiba, Japan. Adaptors were trimmed by fastx_clipper in FASTX-toolkit 0.0.14 (http://hannonlab.cshl.edu/fastx_toolkit/). Nucleotides with QV<10 were trimmed by PRINSEQ 0.20.4 (Schmieder and Edwards, 2011). Filtered single-end reads were mapped predicted genes on the *L. japonicus* MG-20 reference genome (build3.0: https://www.kazusa.or.jp/lotus/) in the end-to-end mode with Bowtie v. 22.1 (Langmead *et al.*, 2009). Raw read counts were normalized to Reads per Kilobase per Million (RPKM) to assess gene expression.

### Accession number

RNA-seq data for the nodules of *L. japonicus* MG-20 inoculated with the USDA61 wild type and NopM knockout strain are available in the DDBJ sequence read archive under Accession No. DRA008355 with Experiment numbers DRX168408 and DRX168409, respectively.

## Results

### Homology of NopM to bacterial T3Es and functional domain predictions

**Fig. 1.**
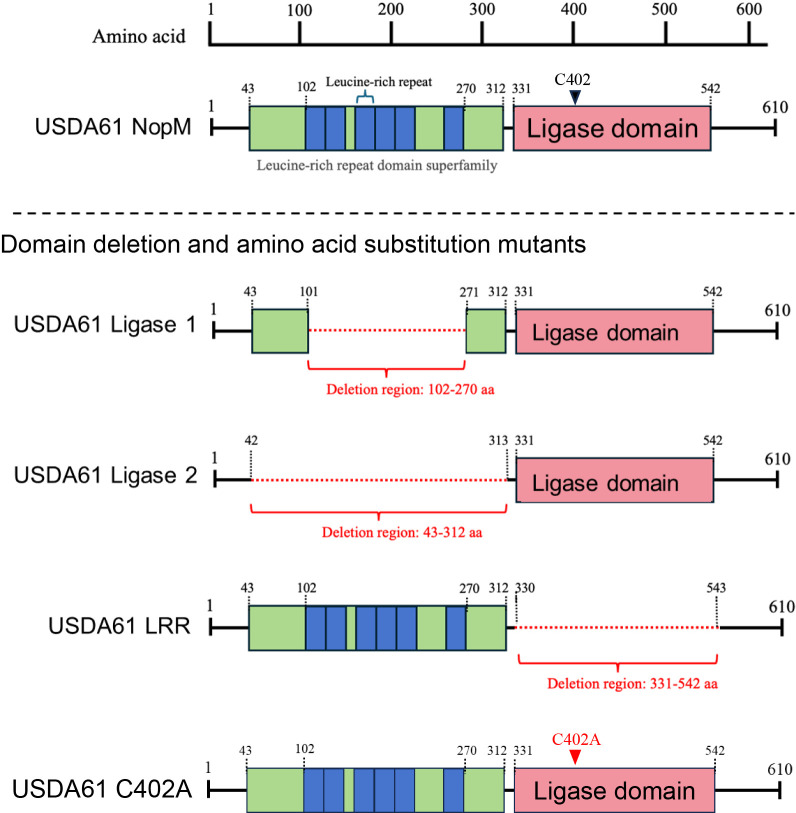
Domain predictions of NopM and its mutants. The domain structure of NopM was analyzed using InterProScan based on its amino acid sequence. Deletion or amino acid substitution mutants were designated as Ligase 1, Ligase 2, LRR, and C402A.

NopM from USDA61 shared 57.6% sequence identity with NopM from *Sinorhizobium* sp. strain NGR234, and 36.6 and 36.9% identities with the E3 ubiquitin ligase (T3Es) Ipa7.8 from *Shigella* and SspH2 from *Salmonella*, respectively (Fig. S1). An ana­lysis of the amino acid sequence of NopM in USDA61 using InterPro predicted that amino acid residues 43 to 312 belonged to the LRR domain superfamily, with six LRRs spanning residues 102 to 270 (Fig. 1). Additionally, residues 331 to 542 were predicted to form a Novel E3 Ligase domain (Fig. 1). The ligase domain contained the catalytic cysteine residue (Cys402), which served as the active center of the ligase and was the only cysteine residue present in the entire NopM protein (Fig. 1 and S1). Based on these structural predictions, we generated domain deletion mutants for the LRR region (residues 102 to 270), the LRR domain superfamily region (residues 43 to 312), and the Novel E3 Ligase domain region (residues 331 to 542) to investigate the function of the NopM domains (Fig. 1). In addition, we generated a mutant in which the cysteine residue at position 402 was substituted with alanine. These mutants were named Ligase 1 (deletion of residues 102 to 270 in the LRR region), Ligase 2 (deletion of residues 43 to 312 in the LRR domain superfamily region), LRR (deletion of residues 331 to 542 in the Novel E3 Ligase domain region), and C402A (Fig. 1).

### Analysis of the secretion of domain-deleted *NopM*

**Fig. 2.**
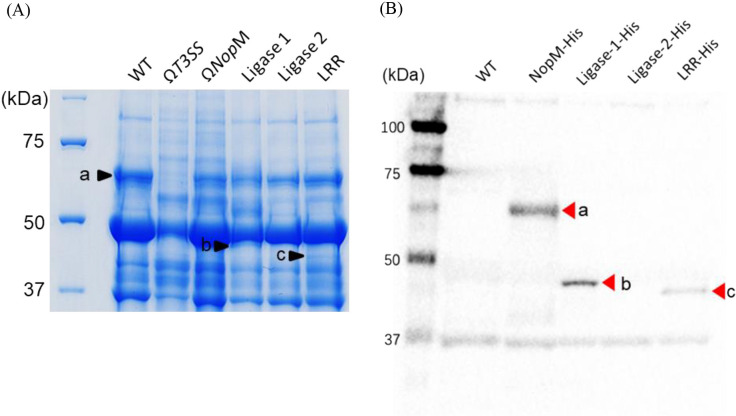
Analysis of secretion into the culture supernatant of domain-deleted NopM. SDS-PAGE ana­lysis of culture supernatant proteins from domain deletion mutants (A). Culture supernatant proteins from each strain were collected and separated by SDS-PAGE, followed by staining with Coomassie Brilliant Blue. Bands a, b, and c correspond to the expected positions of full-length NopM, Ligase 1, and LRR, respectively (A). Detection of His-tagged NopM secretion by Western blotting (B). Plasmids encoding His-tagged NopM and its domain deletion variants were introduced into USDA61. The culture supernatant proteins of these strains were collected, separated by SDS-PAGE, and transferred to a PVDF membrane. A Western blot ana­lysis with an anti-His antibody was performed. Bands a, b, and c correspond to the expected positions of His-NopM, His-Ligase 1, and His-LRR, respectively (B).

The partial deletion of T3Es may affect their secretion via the T3SS; therefore, we collected proteins from the culture supernatants of the generated deletion mutants and analyzed the secreted proteins by SDS-PAGE. In the culture supernatant of the wild-type USDA61 strain, a band corresponding to NopM was detected at the expected mole­cular weight (67.0‍ ‍kDa), as reported in previous studies (Kusakabe *et al.*, 2020) (Fig. 2A). An ana­lysis of the culture supernatants from the deletion mutants revealed that specific bands were detected at the expected mole­cular weights for Ligase 1 (48.6‍ ‍kDa) and LRR (42.7‍ ‍kDa) mutants, respectively (Fig. 2A). In contrast, specific bands were not detected in the culture supernatant lane for the Ligase 2 mutant (Fig. 2A). An ana­lysis of the cysteine substitution mutants was not conducted because secretion was confirmed in the LRR (ligase domain deletion mutant).

To further confirm secretion, we introduced fusion genes of full-length NopM and domain-deleted NopM with a His-tag into USDA61 and extracted proteins from the culture supernatants of these strains. A Western blot ana­lysis using an anti-His tag antibody revealed specific bands at the expected mole­cular weights (67.8‍ ‍kDa for His-NopM full-length, 49.5‍ ‍kDa for His-Ligase 1, and 43.5‍ ‍kDa for His-LRR) in the corresponding lanes (Fig. 2B). In contrast, no specific bands were detected at the expected positions in the culture supernatant lane for the Ligase 2 mutant (Fig. 2B).

### Symbiotic phenotypes of nopM domain deletion mutants

**Fig. 3.**
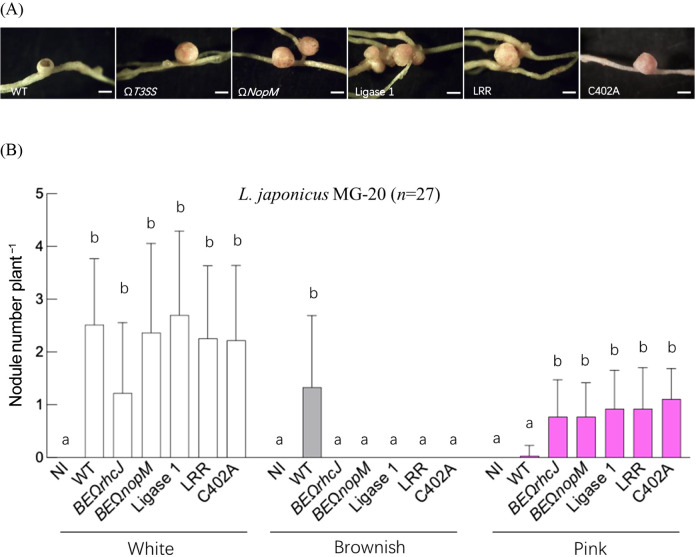
Nodulation phenotypes of NopM domain deletion or amino acid substitution mutants in *Lotus japonicus* MG-20. Nodule images (A) and the number of nodules classified by color (B) were observed and quantified four weeks post-inoculation. NI (non-inoculated) serves as a control group. A statistical ana­lysis of nodule number comparisons (B) was performed using a two-way ANOVA.

To investigate the effects of NopM domain deletions and amino acid substitution on symbiosis with *Lotus* spp., we used Ligase 1, LRR (which had been confirmed to secrete the protein into the culture supernatant), and C402A mutant strains. As previously reported, an inoculation with the USDA61 wild-type strain on *L. japonicus* MG-20 led to the formation of only white and brownish nodules, while pink nodules, indicative of effective symbiosis, were absent (Fig. 3) (Kusakabe *et al.*, 2020). In contrast, the NopM knockout strain, similar to the T3SS knockout strain, significantly reduced the formation of brownish nodules and promoted the development of pink nodules, suggesting that NopM disrupted normal symbiotic development. When the Ligase 1, LRR domain deletion, and C402A mutants were inoculated into MG-20, they exhibited a similar symbiotic phenotype to that of the NopM and T3SS knockout strains (Fig. 3).

**Fig. 4.**
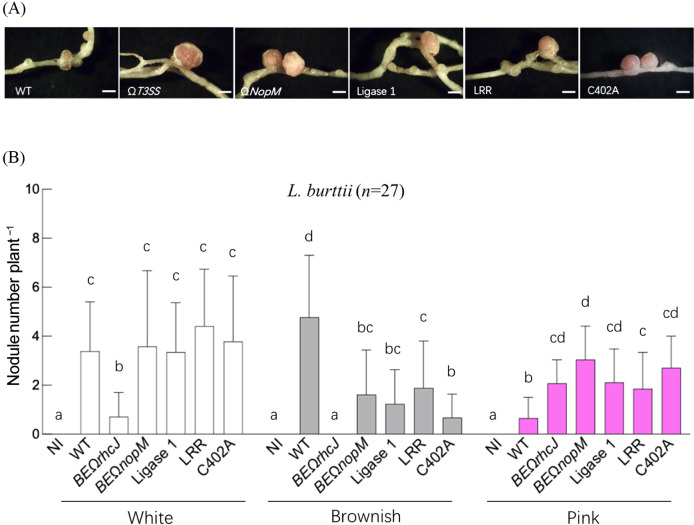
Nodulation phenotypes of NopM domain deletion or amino acid substitution mutants in *Lotus burttii*. Nodule images (A) and the number of nodules classified by color (B) were observed and quantified four weeks post-inoculation. NI (non-inoculated) serves as the control group. A statistical ana­lysis of nodule number comparisons (B) was performed using a two-way ANOVA.

In *L. burttii*, an inoculation with the NopM knockout strain resulted in a lower number of brownish nodules than WT, but a higher number than in plants inoculated with the T3SS knockout strain (Fig. 4). This result suggests that other T3SS effectors, in addition to NopM, contributed to nodule development. An inoculation with the Ligase 1, LRR, and C402A mutants resulted in fewer brownish nodules and more pink nodules than WT, showing a similar phenotype to that of the *NopM* knockout strain (Fig. 4).

### Symbiotic phenotype of *L. japonicus* roots expressing NopM under the control of the leghemoglobin promoter

To further investigate the potential impact of NopM on *L. japonicus* nodule development, we attempted to generate transgenic MG-20 roots constitutively expressing *NopM* under the ubiquitin promoter using *R. rhizogenes*. However, the transformation efficiency of *nopM* was markedly lower than that of the empty vector (EV) control (data not shown). Therefore, we considered it necessary to restrict the expression of *nopM* to nodules and decided to fuse *nopM* to the leghemoglobin promoter, which is specifically expressed in nodules, and introduce it into the roots of *L. japonicus* MG-20.

**Fig. 5.**
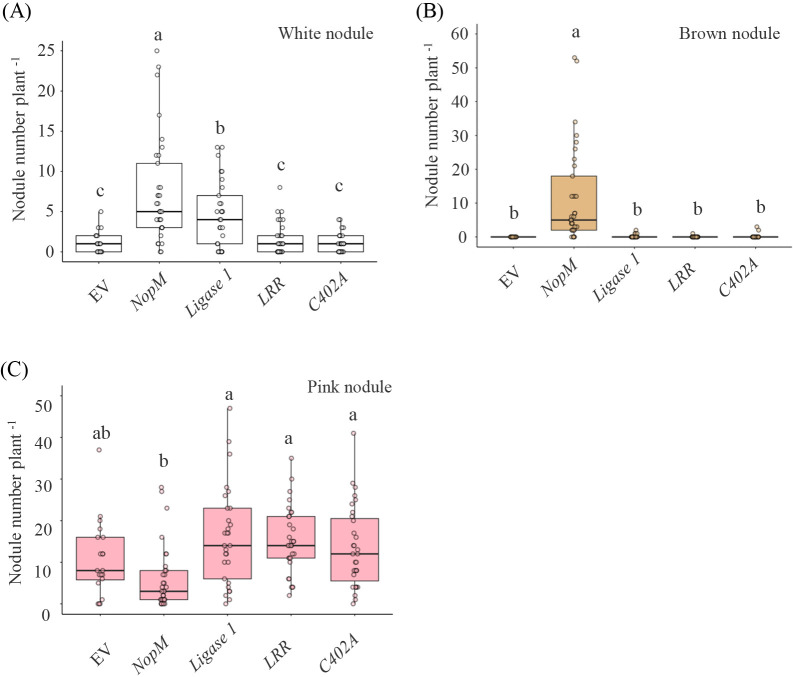
Nodule phenotypes with *Mesorhizobium japonicum* MAFF303099 in transgenic roots of *Lotus japonicus* MG-20 expressing *nopM* and its variants under the control of the LB3 promoter. Transgenic roots were inoculated with rhizobia, and nodule phenotypes were observed 4–5 weeks after the inoculation (35 days post-inoculation in the first replicate, 30 days in the second, and 29 days in the third). The numbers of white, pink, and brown nodules plant^–1^ were counted (A, B and C). *n*=20 (EV), *n*=33 (*NopM*), *n*=29 (*Ligase 1*), *n*=31 (*LRR*), and *n*=31 (*C402A*). The horizontal line within each box shows the median, the bottom and top of the box show the first and third quartiles, and vertical lines extending from the box show the range excluding outliers. Different letters indicate significant differences among treatments as assessed by a one-way ANOVA followed by Tukey’s multiple comparison test (*P*<0.05). EV, empty vector.

A gene in which *nopM* was fused to the *LB3* promoter was introduced into the roots of *L. japonicus* MG-20, and the resulting transformed roots were inoculated with *M. japonicum* MAFF303099. Four to five weeks after the inoculation, nodules exhibiting GFP fluorescence attached to each plant were selected, and the numbers of pink, brown, and white nodules were counted (a representative example is shown in Fig. S2). Roots introduced with *nopM* showed significantly more white and brown nodules (Fig. 5A and B) and fewer pink nodules (Fig. 5C) than EV. In contrast, the introduction of *Ligase 1*, *LRR*, or *C402A* resulted in fewer brown and white nodules, similar to EV (Fig. 5A and B), and more pink nodules than with the introduction of *nopM* (Fig. 5C).

### RNA-seq

To elucidate the mole­cular mechanisms underlying the early senescence-like response in *L. japonicus* nodules induced by USDA61 NopM, we conducted RNA-seq on nodules from the roots of *L. japonicus* MG-20 inoculated with the USDA61 wild type or a NopM-deficient mutant. Total RNA was extracted from the root with nodules four weeks after the inoculation, when brownish nodules were observed. Among the 39,734 genes analyzed, 967 exhibited at least a 2.5-fold up-regulation in response to NopM, whereas 1,603 were up-regulated by at least 2.5-fold in the absence of NopM.

Among the top 100 genes that were up-regulated by at least 2.5-fold in the absence of NopM, several played essential roles in nitrogen fixation (Table S1). These included Lj5g3v0465970 (fold change: 254.9), which encodes leghemoglobin, a key protein involved in regulating oxygen partial pressure within nodules, and Lj1g3v3690250 (fold‍ ‍change: 166.0), which encodes homoaconitate synthase, an enzyme essential for the biosynthesis of the iron-molybdenum cofactor at the active center of nitrogenase (Table S1). These results suggest that normal nodule maturation progressed in the absence of NopM.

Many of the top 100 genes up-regulated by NopM were‍ ‍found to encode proteins with kinase domains or to be‍ ‍associated with plant defense responses (Table S1). In‍ ‍root and nodules inoculated with the USDA61 wild type, the expression levels of the pathogen-related genes *PR1* (Lj1g3v4669290.1) and *PR2* (Lj0g3v0278459.1) were significantly higher, by 1,112.7- and 34.1-fold, respectively, than those inoculated with the *nopM*-deficient mutant (Table S1). In addition, the expression of several *WRKY* transcription factors (Lj0g3v0242439.1, Lj0g3v0130569.1, Lj0g3v0074419.1, and Lj0g3v0330909.1), which are known to play roles in regulating immune and stress responses, was also increased. Furthermore, genes encoding cytochrome P450 enzymes involved in phytoalexin biosynthesis (Lj0g3v0180559.1, Lj6g3v1415840.1) and those predicted to be involved in Ca^2+^ transport (Lj0g3v0050429 and Lj6g3v2118960), which plays a critical role in the early stages of the hypersensitive response (Levine *et al.*, 1996), were up-regulated.

## Discussion

We previously demonstrated that the presence of NopM, a predicted E3 ubiquitin ligase and a type III effector of *B. elkanii* USDA61, induced the formation of incomplete brown nodules (early senescence-like nodules) during symbiosis with *L. japonicus* MG-20 and *L. burttii* (Kusakabe *et al.*, 2020). In the present study, to elucidate the mechanisms by which NopM induces brown nodule formation, we investigated the effects of domain deletions and amino acid substitutions in *nopM* of USDA61 on symbiotic phenotypes. Furthermore, we approached the phenomenon defined as the early senescence-like response induced by NopM through a transcriptome ana­lysis.

NopM of USDA61 is annotated as an E3 ubiquitin ligase and shares 36.6, 36.9, and 57.6% protein identities with the‍ ‍type III effectors of IpaH7.8 in *Shigella*, SspH2 in *Salmonella*, and NopM in *Sinorhizobium* sp. NGR234, respectively (Fig. S1). Pathogenic IpaH7.8 exerts its function by interacting with host target proteins via its N-terminal LRR domain and ubiquitinating them through its C-terminal ligase domain (Suzuki *et al.*, 2014). In addition, although the function of the LRR domain in NopM from NGR234 remains unclear, its ligase activity, mediated by a cysteine residue in the ligase domain, has been reported to affect root nodule symbiosis in a legume host-dependent manner (Xin *et al.*, 2012; Xu *et al.*, 2018). Since USDA61 NopM also possesses a conserved N-terminal LRR domain, C-terminal ligase domain, and cysteine residue within the ligase domain (Fig. 1 and S1), we hypothesized that it may function through a similar mechanism as its pathogenic counterparts. To investigate this, we generated *nopM* mutants lacking the LRR or ligase domain or with the substitution of a cysteine at position 402 (the putative ligase catalytic center) to alanine and analyzed their symbiotic phenotypes. Using Ligase 1 and LRR strains, which were confirmed to be secreted into the culture supernatant (Fig. 2 and 3), we exami­ned their symbiotic phenotypes with *L. japonicus* MG-20 and *L. burttii*. Both of the domain deletion mutants exhibited a reduced number of brown nodules and an increased number of pink nodules, similar to the NopM knockout mutant (Fig. 3 and 4). In addition to these mutants, the mutant in which the cysteine at position 402 was substituted with alanine also acquired a similar symbiotic ability to the *NopM* knockout mutant (Fig. 3 and 4). Furthermore, we generated the transformed roots of *L. japonicus* expressing NopM under the control of the LB3 promoter and exami­ned their symbiotic phenotype with *M. japonicum* MAFF303099. A significant increase in brown nodules was observed upon the introduction of NopM (Fig. 5B). In contrast, in roots transformed with *nopM* lacking each domain or with the cysteine at position 402 substituted with alanine, almost no brown nodules formed (Fig. 5B). These results indicate that the LRR and ligase domains of NopM were both essential for inducing brown nodules in *Lotus* spp., and also that these two domains functioned cooperatively to induce brown nodules. Moreover, since the cysteine residue in the ligase domain, which is predicted to be the catalytic site for ligase activity, was essential for the induction of brown nodules, these results strongly suggest that NopM functions as an E3 ubiquitin ligase, similar to those found in pathogenic bacteria and rhizobia. In transformed root experiments, previous studies demonstrated that the introduction of *nopM* from *Sinorhizobium* sp. NGR234 into *L. japonicus* MG-20 induced the formation of smaller nodules than EV, and this phenotype was not observed when an inactivated mutant (C338A) was introduced (Xu *et al.*, 2018). In the present study, the introduction of NopM derived from USDA61 also resulted in the formation of smaller nodules (brown) than the pink nodules in EV (Fig. S2), and this phenotype was scarcely observed with the inactivated mutant *nopM* (C402A). Although nodule colors were not described in the previous study (Xu *et al.*, 2018), the formation of smaller nodules upon the introduction of *nopM* was consistently observed. These results suggest that USDA61 NopM, similar to NGR234 NopM, negatively affected nodule development in *L. japonicus* through its E3 ubiquitin ligase activity. Therefore, we propose that USDA61 NopM interacts with host factors via its LRR domain and ubiquitinates them through its ligase domain, and also that this activity contributes to the induction of brown nodules in *L. japonicus* and *L. burttii*.

Furthermore, RNA-seq revealed that the expression of several immune-related genes, including PR proteins, WRKY transcription factors, and cytochrome P450 enzymes involved in phytoalexin biosynthesis, were up-regulated in response to NopM. In addition, genes associated with Ca^2+^ transport, which plays an important role in early HR responses (Levine *et al.*, 1996), were up-regulated. The brown coloration induced by *nopM* was only observed in the nodules, not in the root (Fig. 3A and 4A). In addition, in many cases, the brown coloration was observed in a site-specific manner within a single nodule (Fig. 3A and 4A). Collectively, these results suggest that the brown nodules represent a physiological response very similar to a hypersensitive cell death reaction that localizes pathogen infection through defense responses and site-specific cell death. Therefore, although NopM was previously reported to induce an early senescence-like response in *L. japonicus* and *L. burttii* nodules, the present results strongly suggest that the response induced by NopM closely resembles a hypersensitive response.

In the present study, we conducted a detailed investigation of the function of NopM, a type III effector (T3E) derived from USDA61, which induces brown nodules in *L. japonicus* and *L. burttii* and causes symbiosis failure. The results obtained suggest that NopM specifically ubiquitinated target proteins through its LRR and ligase domains, leading to the formation of brown nodules. Furthermore, the results of the transcriptome ana­lysis strongly indicate that the early senescence-like response induced by NopM within the nodules is a form of hypersensitive cell death. Since plant pathogens progress infection via E3 ubiquitin ligases (Nakano *et al.*, 2017), *L. japonicus* may possess a system that recognizes pathogen-derived E3 ligases and triggers hypersensitive cell death to eliminate these pathogens. This defense mechanism may also function against rhizobia that have acquired E3 ligase genes through horizontal gene transfer from pathogens. An important future challenge is to identify the specific target proteins of NopM, which is crucial for understanding the mole­cular basis of this immune response. In the present study, we demonstrated that the LRR domain, which is considered to be important for interactions with targets, was essential for the function of NopM; however, identifying key amino acid residues within the LRR domain warrants further study. Moreover, through cysteine substitution experiments, we demonstrated that ligase activity was important for the function of NopM; however, confirming the presence or absence of ubiquitin ligase activity and substrate specificity both *in vitro* and *in vivo* remains a future challenge. Additionally, a recent preprint reported that the introduction of *nopM* from *Sinorhizobium* sp. NGR234 into *L. japonicus* increased the number of infection threads of *M. japonicum* (Wang *et al.*, 2024). While the present study focused on nodule phenotypes, the effects of introducing *nopM* on the infection process also warrants further investigation. By addressing these issues, we will clarify the mechanisms by which this effector affects plant immune signaling pathways. These insights may contribute to the development of sustainable agricultural technologies that harness microbes by regulating immune responses during symbiosis and manipulating host specificity between leguminous plants and rhizobia.

## Citation

Ying, C., Nozawa, S., Kusakabe, S., Songwattana, P., Piromyou, P., Boonchuen, P., et al. (2025) The Type III Effector NopM from *Bradyrhizobium elkanii* USDA61 Induces a Hypersensitive Response in *Lotus japonicus* Root Nodules. *Microbes Environ ***40**: ME25020.

https://doi.org/10.1264/jsme2.ME25020

## Supplementary Material

Supplementary Material

## Figures and Tables

**Table 1.** Bacterial strains and plasmids used in the present study

Strains or Plasmids	Characteristics^a^	Reference or Source
***Bradyrhizobium elkanii***		
USDA61	Wild-type strain, Pol^r^	Keyser (United States Department of Agriculture, Beltsville, MD)
BErhcJ	USDA61 derivative harboring an insertion in the *rhcJ* region; Pol^r^, Km^r^, Tc^r^	Okazaki *et al.*, 2009
BEnopM	USDA61 derivative harboring an insertion in the *nopM* region; Pol^r^, Km^r^, Tc^r^	Kusakabe *et al.*, 2020
Ligase1	USDA61 derivative with deletion in the nopM LRR domain (102–270); Pol^r^	This study
Ligase2	USDA61 derivative with deletion in the nopM LRR superfamily (43–312); Pol^r^	This study
LRR	USDA61 derivative with deletion in the nopM novel E3 ligase domain (331–542); Pol^r^	This study
BenopM (C402A)	USDA61 derivative, substitution of the cysteine at position 402 of NopM with alanine; Pol^r^	This study
***Mesorhizobium japonicum***		
MAFF303099	Wild-type strain; Pm^r^	Saeki and Kouchi, 2000
***Rhizobium rhizogenes***		
AR1193	Used for *L. japonicus* hairy root transformation	Hansen *et al.*, 1989
***Escherichia coli***		
DH5α	Cloning host; *F*-Φ80dlacZΔM15 Δ(lacZYA-argF) U169 deoR recA1 endA1 hsdR17(rK^–^ mK^+^) phoA supE44 λ^–^ thi-1 gyrA96 relA1	Takara Bio
**Plasmids**		
pRK2013	Helper strain carrying RK2 transfer genes; Km^r^, *tra*	Figurski and Helinski, 1979
pK18mobsacB	Suicide vector for allelic exchange; *sacB*, Km^r^	Schäfer *et al.*, 1994
pK18mobsacB_Ligase1	pK18mobsacB derivative for the *nopM* LRR deletion (amino acids 102–270); *sacB*, Km^r^	This study
pK18mobsacB_Ligase2	pK18mobsacB derivative for the *nopM* LRR superfamily deletion (amino acids 43–312); *sacB*, Km^r^	This study
pK18mobsacB_LRR	pK18mobsacB derivative for the *nopM* E3 ligase deletion (amino acids 331–542); *sacB*, Km^r^	This study
pK18mobsacB_NopM±700 bp	pK18mobsacB containing *nopM* of USDA61 with 700-bp upstream and downstream regions; *sacB*, Km^r^	This study
pK18mobsacB_C402A	pK18mobsacB derivative for substitution of the cysteine 402 of NopM with alanine; *sacB*, Km^r^	This study
pK18mobsacB_Ligase1_His	pK18mobsacB derivative for *nopM* Ligase-1-His expression in USDA61; *sacB*, Km^r^	This study
pK18mobsacB_Ligase2_His	pK18mobsacB derivative for *nopM* Ligase-2-His expression in USDA61; *sacB*, Km^r^	This study
pK18mobsacB_LRR_His	pK18mobsacB derivative for *nopM* LRR-His expression in USDA61;* sacB*, Km^r^	This study
pUB-GFP	Binary vector for hairy root transformation, 35S promoter-driven GFP; Km^r^	Maekawa *et al.*, 2008
pCambia_Lb3promoter_GW_Lb3terminator (GFP)	Binary vector with *L. japonicus* LB3 promotor and terminator	Shimoda *et al.*, 2019
pLB3-NopM	pUB-GFP derivative, the ubiquitin promoter and terminator were removed and the LB3 promoter-NopM-LB3 terminator was introduced.	This study
pLB3-Ligase 1	pUB-GFP derivative, the ubiquitin promoter and terminator were removed and the LB3 promoter-Ligase 1-LB3 terminator was introduced.	This study
pLB3-LRR	pUB-GFP derivative, the ubiquitin promoter and terminator were removed and the LB3 promoter-LRR-LB3 terminator was introduced.	This study
pLB3-C402A	pUB-GFP derivative, the ubiquitin promoter and terminator were removed and the LB3 promoter-NopM(C402A)-LB3 terminator was introduced.	This study

Pol^r^, polymyxin resistant; Km^r^, kanamycin resistant; Pm^r^, phosphomycin resistant; Tc^r^, tetracycline resistant.

**Table 2.** Primers used in the present study

Primer name	Sequence (5′-3′)	Usage
Ligase1_1F	TCGAGCTCGGTACCCTCTGGCAAGATCGATCC	USDA61 Ligase 1 construction
Ligase1_1R	AGAATAACCGCTTAGGGTCGACGCAGGCAAGC	
Ligase1_2F	TTGCCTGCGTCGACCCTAAGCGGTTATTCTAGC	
Ligase1_2R	CTCTAGAGGATCCCCCGAGGACCTGATGCATGACC	
Ligase2_1F	TCGAGCTCGGTACCCCGGTGCTCGAGTTTA	USDA61 Ligase 2 construction
Ligase2_1R	GCGCCGCCAGCGGACGCCAG	
Ligase2_2F	GTCCGCTGGCGGCGCGCTGGAAGTC	
Ligase2_2R	CTCTAGAGGATCCCCCATTCGCTCGACGAAACTTCTCC	
LRR_1F	TCGAGCTCGGTACCCAGAGACGGCGATGAATAC	USDA61 LRR construction
LRR_1R	GCAACATGGTCGAGCCAGTGCGCCAC	
LRR_2F	TGGCGCACTGGCTCGACCATGTTGCAATG	
LRR_2R	CTCTAGAGGATCCCCCGGAGACCGCAGATG	
NopM_up700_F	TCGAGCTCGGTACCCCGATCTGGCAAGATC	pK18mobsacB_NopM±700 bp construction
NopM_dw700_R	CTCTAGAGGATCCCCGCTTCCACGCCTTCG	
C402A_F	GAGCGCGCCGAAGATCGGGTTACCTTGACC	USDA61 C402A construction
C402A_R	ATCTTCGGCGCGCTCGCTCGCTCC	
pK18_nopM_His6_F	TCGAGCTCGGTACCCCTCGGGAGTAGGAGCCG	Introduction of His-tagged NopM into *B. elkanii*.
pK18_nopM_His6_R	GACTCTAGAGGATCCCCTCAGTGGTGGTGGTGGTGGTGAAGCTCAAGTCCGAATC	
LB3pro_nopM61_F	GCGCCCACCCTTTTATCAAAGCTCAAGTCCGA	Introduction of *nopM* and its derivatives into *L. japonicus* roots
LB3ter_nopM61_R	GCCGCCCCCTTCACCATGAATACAGAGCAG	
NopM61_CtoA_R	GCGAGCGAGCGCGCCGAAGATCGGGTTACCTTGAC	
NopM61_CtoA_F	GGCGCGCTCGCTCGCTCCCGAGGCCAGTTG	
Inverse_pUB-GFP_without_UbiProTer_F	CGAATTCCTGCAGCCCAG	
Inverse_pUB-GFP_without_UbiProTer_R	GAGCTCGAATTCGTAATCATGGTCA	
LB3_promotor_F	GGCTGCAGGAATTCGCTCGAGATATCCCATGCACC	
LB3_promotor_R	GGTGAAGGGGGCGGCCGCGG	
LB3_terminator_F	TACGAATTCGAGCTCCACTTTCATGATTGCAGTTGTTTG	
LB3_terminator_R	TAAAAGGGTGGGCGCGCCGACCCAGC	
