# Supplementary material for: The Type III Effector NopM from Bradyrhizobium elkanii USDA61 Induces a Hypersensitive Response in Lotus japonicus Root Nodules
Source: Microbes Environ. 2025 Oct 15;40(4):ME25020. doi: 10.1264/jsme2.ME25020 (PMC12727206; doi:10.1264/jsme2.ME25020)
Supplement: Supplementary file 1 — Supplementary Material [file 40_25020_s1.pdf]

Table S1. Top 100 genes with increased expression under the presence of NopM

GeneID	Fold change	Annotation	Classification
Lj1g3v4669290.1	1112.7	Thaumatin family,Thaumatin, pathogenesis-related	PR
Lj0g3v0242439.1	84.0	WRKY transcription factor	WRKY
Lj5g3v2013610.1	80.4	Un Characterized	
Lj0g3v0130569.1	80.2	WRKY transcription factor	WRKY
Lj0g3v0180559.1	50.7	Flavonoid 3'-hydroxylase ,Cytochrome P450	Cytochrome P450
Lj0g3v0306109.1	42.5	Predicted protein	
Lj0g3v0331979.1	36.9	Uncharacterized protein,Protein kinase-like (PK-like),Protein kinase-like domain	kinase
Lj0g3v0278459.1	34.1	Glycoside hydrolase	PR
Lj0g3v0294819.3	30.0	Putative uncharacterized protein, Protein kinase-like (PK-like),Protein kinase-like domain	kinase
Lj4g3v2604510.1	29.3	Putative copper ion-binding protein	
Lj3g3v0563880.1	28.4	Uncharacterized protein	
Lj0g3v0074419.1	26.8	WRKY,DNA-binding WRKY; WRKY DNA-binding domain,DNA-binding WRKY	WRKY
Lj0g3v0272009.1	26.5	Non-specific lipid-transfer protein (Precursor)	
Lj0g3v0101099.1	24.4	Dimethylaniline monooxygenase	
Lj0g3v0249229.1	23.5	Uncharacterized protein	
Lj2g3v1550130.1	23.3	Chloramphenicol acetyltransferase-like domain	
Lj4g3v1880320.1	21.8	Cysteine-rich receptor-like protein kinase	
Lj0g3v0050429.1	21.5	Cation-transporting atpase	Ca
Lj5g3v1073800.1	21.3	Receptor like kinase	kinase
Lj5g3v1961260.1	21.0	Glycoside hydrolase, family 18, catalytic domain	PR
Lj6g3v1078490.1	20.8	PLC-like phosphodiesterase	
Lj2g3v1199960.1	20.8	Putative uncharacterized protein	
Lj1g3v1911780.1	19.1	Kinase-like protein	kinase
Lj0g3v0103129.1	18.9	Uncharacterized protein	
Lj4g3v2916530.1	17.6	Thaumatin-like protein PR-5a (Precursor)	PR
Lj6g3v0325210.1	17.4	Non-specific lipid-transfer protein (Precursor)	
Lj4g3v0098320.1	17.3	Predicted protein	
Lj0g3v0328919.1	17.3	Uncharacterized protein	

Lj2g3v3320570.1	17.3	Kinase-like protein	kinase
Lj3g3v3260400.1	17.1	Uncharacterized protein	
Lj0g3v0330909.1	16.9	Transcription factor, WRKY57	WRKY
Lj4g3v1880270.1	16.9	Receptor like kinase	kinase
Lj1g3v2609220.1	16.3	Non Characterized	
Lj4g3v3099340.1	16.0	MYB transcription factor MYB184	TF
Lj3g3v2662000.1	15.9	Neurofilament heavy polypeptide	
Lj0g3v0176099.1	15.6	Non Characterized	
Lj0g3v0257879.1	15.4	LRR receptor-like serine/threonine-protein kinase ERL2	kinase
Lj3g3v0273600.1	15.3	Non Characterized	
Lj4g3v3014520.1	15.2	Non Characterized	
Lj4g3v1880310.1	15.1	Protein kinase	kinase
Lj2g3v1644320.1	14.4	NUCLEOPORIN-RELATED,NULL; Dirigent,Plant disease resistance response protein	
Lj1g3v2952270.1	14.2	Uncharacterized protein	
Lj1g3v2098310.1	14.0	Uncharacterized protein	
Lj0g3v0009919.1	14.0	Putative uncharacterized protein	kinase
Lj1g3v1708950.1	13.7	THIHA Uncharacterized protein	
Lj6g3v0040360.1	13.6	Putative uncharacterized protein	
Lj5g3v1999150.1	13.4	Uncharacterized protein	
Lj2g3v3337550.2	12.5	Calcium-transporting ATPase	
Lj0g3v0194019.1	12.0	F-box protein	
Lj6g3v0607050.1	11.6	Polygalactorunase PG11 (Precursor)	
Lj2g3v2772290.1	11.5	Putative uncharacterized protein	
Lj5g3v2241100.1	11.5	Putative uncharacterized protein	kinase
Lj4g3v2819910.1	11.3	Uncharacterized protein	
Lj5g3v2182430.1	11.3	Serine/Threonine protein kinases	kinase
Lj5g3v1073730.1	11.2	Protein kinase-like (PK-like),Protein kinase-like domain	kinase
Lj5g3v2013690.1	11.2	Uncharacterized protein	
Lj0g3v0060419.1	11.0	Uncharacterized protein	
Lj0g3v0130759.1	11.0	Uncharacterized protein	
Lj0g3v0283979.1	10.9	GDSL esterase/lipase	
Lj2g3v1550120.1	10.6	Chloramphenicol acetyltransferase-like domain	
Lj6g3v2118960.1	10.6	CALCIUM-BINDING EF HAND FAMILY PROTEIN	Ca

Lj2g3v3339660.1	10.1	Non Characterized	
Lj3g3v1113000.1	10.1	Metal ion binding protein	
Lj6g3v0227550.1	9.9	Glycoside hydrolase, family 9	PR
Lj0g3v0116329.1	9.8	Uncharacterized protein	
Lj1g3v1821120.1	9.5	Uncharacterized protein	
Lj0g3v0068059.1	9.4	Non Characterized	
Lj0g3v0319879.1	9.4	Non Characterized	
Lj2g3v1415420.1	9.4	Peroxidases heam-ligand binding site	
Lj0g3v0306419.1	9.4	Nodulation pectate lyase	
Lj3g3v3166200.1	9.3	ZINC_FINGER_protein	
Lj0g3v0258259.1	9.3	Non Characterized	
Lj4g3v2604520.1	9.3	heavy metal-associated domain	
Lj0g3v0146639.1	9.2	Pectate lyase	
Lj1g3v0841370.1	9.2	Signal transduction response regulator	
Lj1g3v3329770.1	9.2	RAG1-ACTIVATING PROTEIN 1	
Lj1g3v3207810.1	9.1	F-box,F-box domain, cyclin-like	
Lj6g3v1372100.1	9.1	Uncharacterized protein	
Lj6g3v1415840.1	9.1	Cytochrome P450	Cytochrome P450
Lj2g3v0718100.1	9.0	Calmodulin_bind,Calmodulin binding protein-like	Ca
Lj2g3v1199950.1	9.0	Putative uncharacterized protein	
Lj0g3v0053779.1	9.0	Uncharacterized protein	
Lj3g3v1788950.1	9.0	ATP-dependent DNA helicase PIF1	
Lj0g3v0235239.1	9.0	Thioredoxin-like,Thioredoxin-like fold	
Lj0g3v0188629.1	8.9	ATP,Protein kinase	
Lj0g3v0361439.1	8.9	Putative uncharacterized protein	
Lj0g3v0093759.1	8.8	Uncharacterized protein	
Lj2g3v1079620.1	8.8	UDP-glucuronosyl/UDP-glucosyltransferase	
Lj4g3v1880230.1	8.8	PROTEIN_KINASE_ATP,Protein kinase, ATP binding site	kinase
Lj6g3v1177360.1	8.8	tr|B9VI84|B9VI84_ORYSJ Truncated keratin associated protein	
Lj4g3v2775560.1	8.7	Putative uncharacterized protein	
Lj0g3v0161109.1	8.6	Receptor-like protein kinase	
Lj5g3v0878400.1	8.6	Aquaporin, MIP family, NIP subfamily	
Lj0g3v0229479.1	8.6	Non Characterized	
Lj1g3v1798860.1	8.5	Ankyrin repeat-containing protein	
Lj0g3v0146039.1	8.5	Tyrosine-protein kinase ABL	

Lj0g3v0013429.1	8.4	Expansin
Lj1g3v3318570.1	8.4	Aquaporin, MIP family, NIP subfamily
Lj5g3v2045600.1	8.4	Uncharacterized protein
Lj6g3v0214770.1	8.4	NUDIX hydrolase domain-like

Table S2. Top 100 genes with increased expression under the absence of NopM

GeneID	Fold change	Annotation
Lj4g3v0937820.2	2152.7	Putative polyol transporter 1
Lj5g3v1497840.1	1238.8	Respiratory burst oxidase-like protein
Lj0g3v0091689.1	863.2	Copper transporter
Lj3g3v0139560.2	695.3	Auxin-induced protein 5NG4
Lj5g3v1887360.1	392.7	Uncharacterized protein
Lj0g3v0171589.1	312.4	Non Characterized
Lj2g3v0391820.1	286.3	Non Characterized
Lj5g3v1886260.1	266.0	laccase,Laccase; Cu-oxidase,Multicopper oxidase, type 1
Lj5g3v0465970.1	254.9	Leghemoglobin
Lj0g3v0172799.2	236.0	Non Characterized
Lj4g3v0336000.1	234.9	Drug/metabolite transporter; Multidrug resistance efflux transporter EmrE
Lj2g3v1012910.1	201.9	ZINC FINGER FYVE DOMAIN CONTAINING PROTEIN
Lj2g3v0391810.1	199.0	Non Characterized
Lj0g3v0137939.1	198.1	Non Characterized
Lj4g3v2573630.2	189.6	Transcription factor, MADS-box; coiled-coil
Lj0g3v0357029.1	178.2	ZINC FINGER FYVE DOMAIN CONTAINING PROTEIN
Lj0g3v0325609.1	172.3	NADH-ubiquinone reductase complex 1
Lj1g3v3690250.1	166.0	Homocitrate synthase
Lj2g3v3071890.1	147.7	Non Characterized
Lj3g3v0040860.1	139.5	Non Characterized
Lj5g3v0035290.2	139.4	Non Characterized
Lj3g3v3031030.1	134.3	Non Characterized
Lj5g3v1427660.1	98.8	Non Characterized
Lj6g3v2085690.1	93.3	Predicted protein
Lj6g3v1934080.1	91.8	Potassium channel
Lj0g3v0035419.1	90.9	SWEET sugar transporter
Lj3g3v0323310.1	88.1	Non Characterized
Lj1g3v3976010.1	87.3	RmlC-like cupins,RmlC-like cupin domain; no description,RmlC-like jelly roll fold
Lj0g3v0257439.2	82.5	Cysteamine dioxygenase; no description,RmlC-like jelly roll fold
Lj1g3v3444080.1	73.5	Ferric reductase defective 3b
Lj0g3v0278159.1	65.7	Nodule-specific protein Nlj70

Lj0g3v0247829.1	63.5	Non Characterized
Lj2g3v0776870.1	62.4	Sst1 protein
Lj2g3v0776860.1	62.1	Non Characterized
Lj3g3v3086070.1	62.0	Beta-galactosidase
Lj1g3v3101600.1	58.5	Non Characterized
Lj3g3v2873690.1	58.0	Non Characterized
Lj1g3v2953570.1	54.9	Uncharacterized protein
Lj1g3v1049430.1	49.8	Uncharacterized protein
Lj0g3v0289849.1	43.4	Uncharacterized protein
Lj0g3v0256929.1	41.8	Uncharacterized protein
Lj2g3v1034640.1	41.4	AP2-like ethylene-responsive transcription factor
Lj0g3v0185829.1	41.1	Non Characterized
Lj0g3v0324769.1	40.6	Putative uncharacterized protein
Lj4g3v0338190.1	40.0	PLP-dependent transferases,Pyridoxal phosphate-dependent transferase
Lj5g3v1811660.1	39.7	Pleckstrin homology domain.,Pleckstrin homology domain; in StAR and phosphatidylcholine transfer pro
Lj0g3v0185849.1	37.8	S-adenosyl-L-methionine-dependent methyltransferases
Lj1g3v2994540.1	37.3	Methyltransf_2,O-methyltransferase, family 2; Dimerisation,Plant methyltransferase dimeris
Lj1g3v4693080.1	37.3	Non Characterize
Lj6g3v2218710.1	37.2	,Pyridoxal phosphate-dependent transferase
Lj1g3v1195650.1	36.8	Non Characterized
Lj1g3v4515810.1	35.1	OLIGOPEPTIDE TRANSPORTER-RELATED,NULL; OLIGOPEPTIDE TRANSPORTER-RELATED,Proton-dependent oligopeptid
Lj5g3v0780660.1	34.6	A-type carbonic anhydrase
Lj2g3v1778740.1	33.9	Non Characterized
Lj3g3v2477640.1	33.2	ATP-binding cassette protein
Lj6g3v0815810.1	32.1	Dihydroneopterin aldolase/epimerase domain; folB_dom: FolB domain,Dihydroneopterin aldolase
Lj1g3v3443990.1	31.1	Zinc finger, C2H2; no description,Zinc finger C2H2-type/integrase DNA-binding dom
Lj1g3v3438870.1	30.8	Fructose-1,6-bisphosphatase class 1/Sedoheputulose-1,7-bisphosphatase
Lj2g3v1828680.1	28.8	Non Characterized
Lj2g3v1014120.1	27.2	Non Characterized

Lj0g3v0253119.1	26.8	Peptidase S8, subtilisin, Ser-active site; no description,Peptidase S8/S53
Lj0g3v0308749.1	26.0	ABI3-interacting protein
Lj1g3v0627500.2	25.4	Non Characterized
Lj4g3v2578220.1	25.1	,UDP-glucuronosyl/UDP-glucosyltransferase; no description
Lj2g3v2843610.1	25.1	Predicted protein
Lj3g3v2873680.1	24.7	Non Characterized
Lj0g3v0130479.1	24.6	Non Characterized
Lj4g3v0867510.1	24.5	Non Characterized
Lj2g3v3089600.1	24.2	Non Characterized
Lj5g3v2166250.1	23.8	Cytochrome P450
Lj6g3v1048890.1	23.3	Domain of unknown function
Lj1g3v1596890.1	23.1	Uncharacterized protein
Lj3g3v2920860.1	23.0	Non Characterized
Lj2g3v2017510.1	22.8	Non Characterized
Lj2g3v1758440.1	22.8	Putative polyol transporter protein 2 (Fragment)
Lj4g3v2133900.1	22.5	Non Characterized
Lj0g3v0313169.2	22.0	Non Characterized
Lj3g3v2742950.1	21.7	Rieske [2Fe-2S] iron-sulphur domain; seg,NULL; CytB6-F_Fe-S,Cytochrome b6-f complex F
Lj6g3v0815760.1	21.6	Non Characterized
Lj0g3v0311669.1	21.3	Short-chain dehydrogenase/reductase
Lj1g3v3256680.1	21.2	Non Characterized
Lj1g3v3580540.1	20.8	Tetrahydrobiopterin biosynthesis enzymes-like
Lj1g3v4564980.1	19.8	Multidrug resistance efflux transporter EmrE,NULL; EamA,Drug/metabolite transporter
Lj0g3v0095729.1	19.7	Non Characterized
Lj3g3v0526190.1	19.5	Non Characterized
Lj0g3v0080199.1	19.2	Nodule-specific protein Nlj70
Lj3g3v2873670.1	19.1	Non Characterized
Lj1g3v3779370.1	19.0	Kelch repeat-containing protein (Precursor)
Lj0g3v0214939.1	18.8	Non Characterized
Lj0g3v0302699.1	18.6	UDP-glucuronosyl/UDP-glucosyltransferase; UDP-Glycosyltransferase/glycogen phosphorylase
Lj0g3v0327839.1	18.5	Lytic transglycosylase, catalytic (Precursor)
Lj1g3v5048280.1	18.5	Non Characterized
Lj0g3v0355629.1	18.5	RNA binding protein, putative
Lj5g3v1500430.1	18.1	Phospholipase D/Transphosphatidylase

Lj3g3v0819900.1	18.0	Non Characterized
Lj4g3v0336320.1	18.0	Non Characterized
Lj3g3v0126170.1	18.0	Non Characterized
Lj1g3v4578570.1	18.0	Non Characterized
Lj3g3v0323320.2	17.9	Calmodulin-binding protein-like protein
Lj0g3v0182069.1	17.5	Non Characterized

Ying *et al.*, Fig. S1

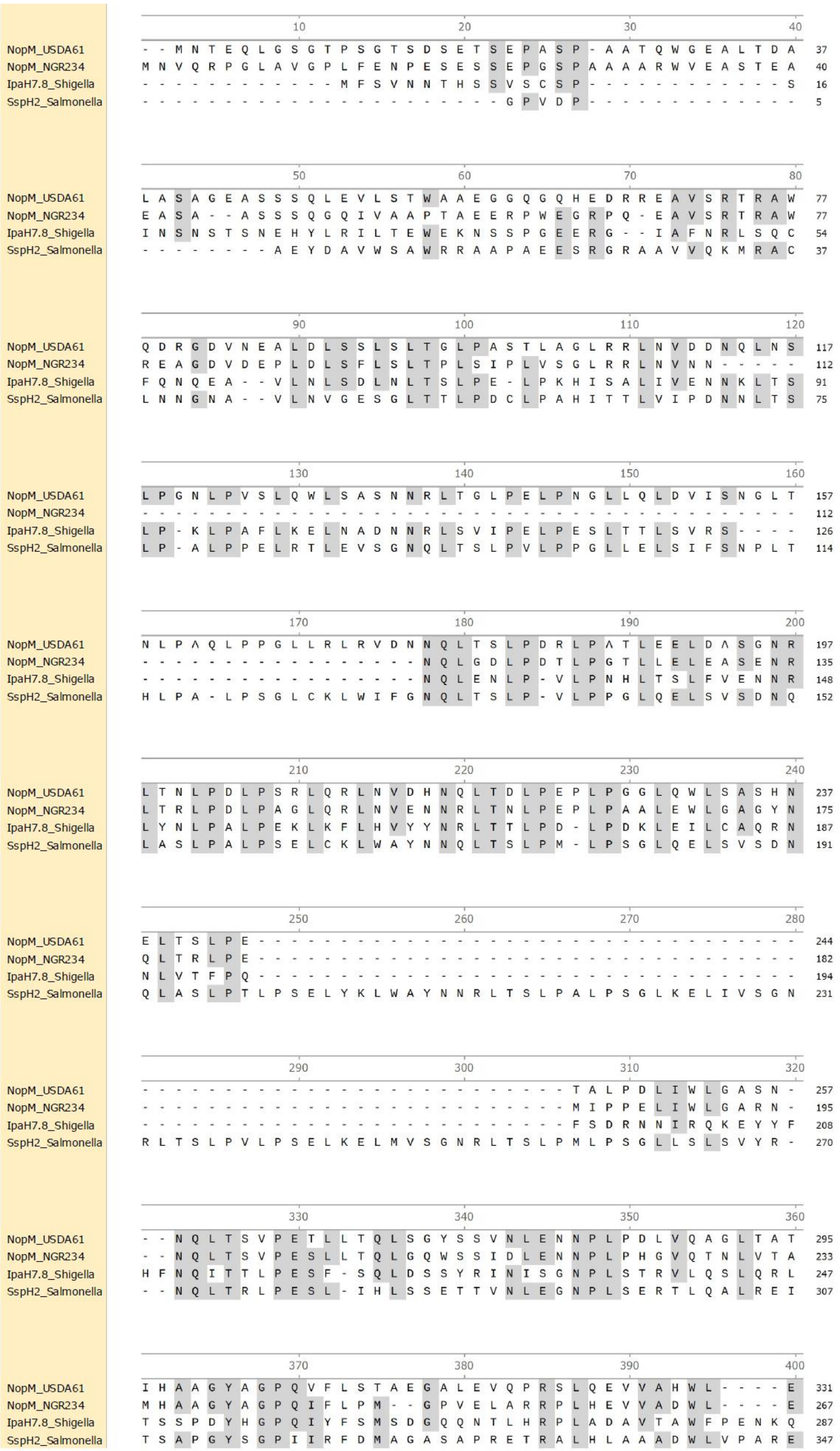

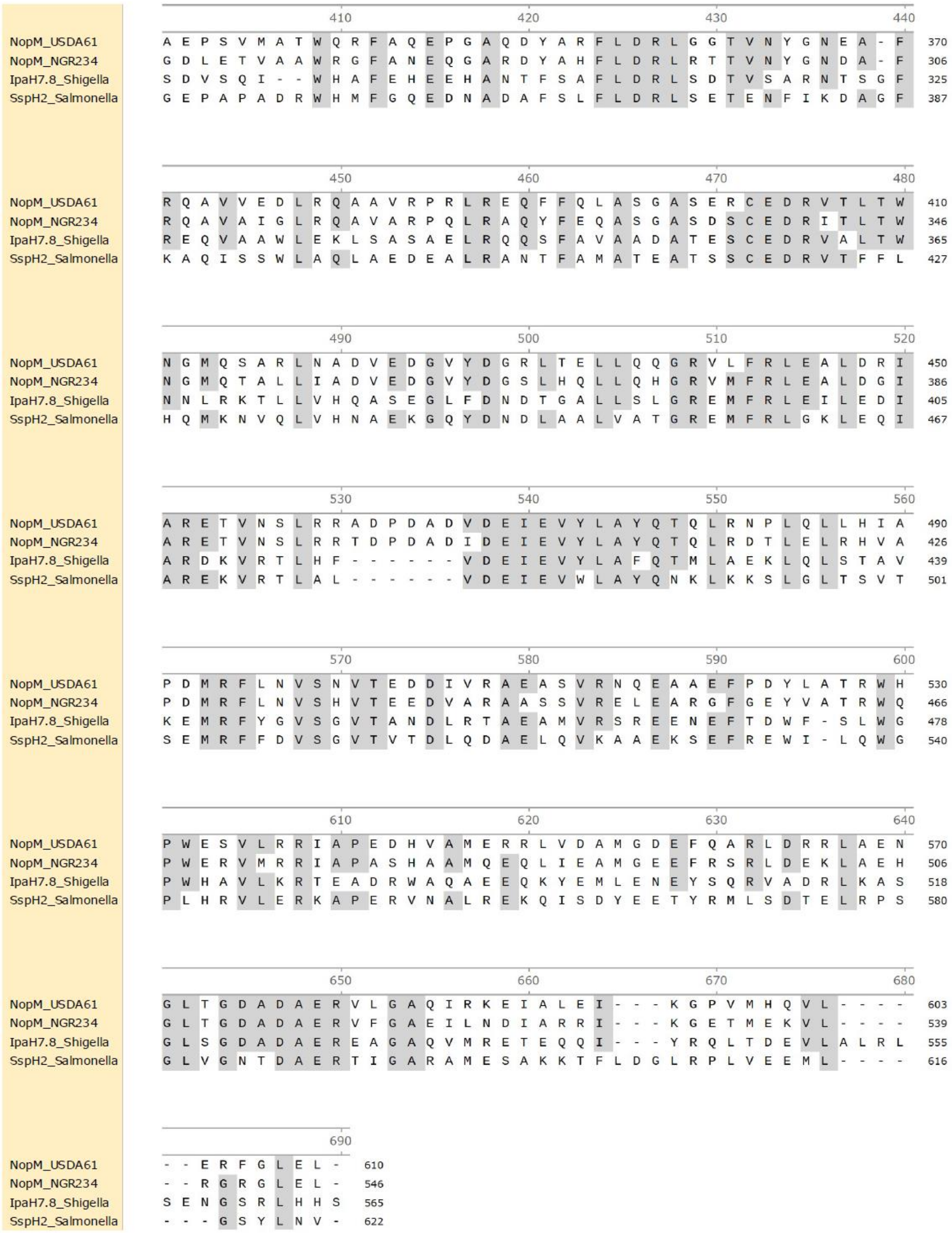

Fig. S1. Amino acid sequence alignment of NopM from *Bradyrhizobium elkanii* USDA61, NopM from *Sinorhizobium* sp. NGR234, IpaH7.8 from *Shigella flexneri*, and SspH2 from *Salmonella typhimurium*.

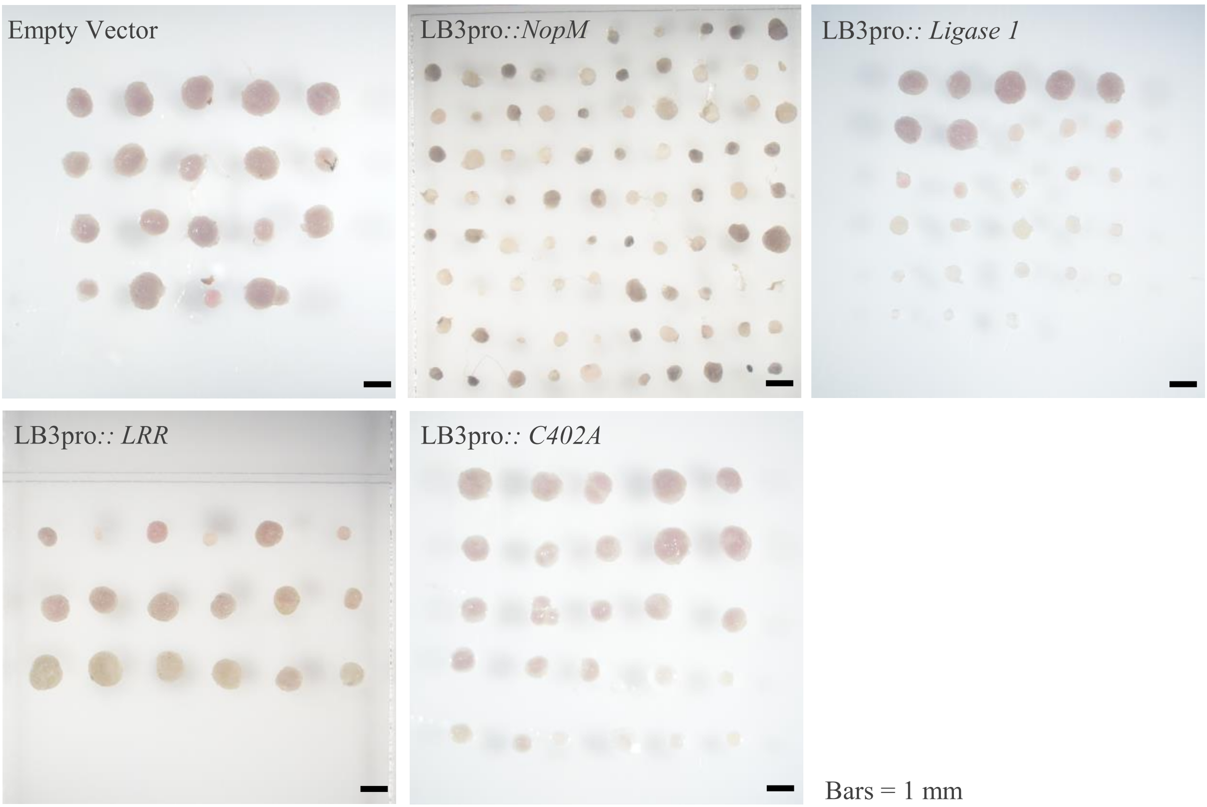

Fig. S2 Representative nodules with *Mesorhizobium japonicum* MAFF303099 on transgenic roots of *Lotus japonicus* MG-20 expressing *nopM* and its variants under the control of the LB3 promoter.
